# The Collagen Synthesis Response to an Acute Bout of Resistance Exercise Is Greater when Ingesting 30 g Hydrolyzed Collagen Compared with 15 g and 0 g in Resistance-Trained Young Men

**DOI:** 10.1016/j.tjnut.2023.10.030

**Published:** 2023-11-24

**Authors:** Joonsung Lee, Jonathan C.Y. Tang, John Dutton, Rachel Dunn, William D. Fraser, Kevin Enright, David R. Clark, Claire E. Stewart, Robert M. Erskine

**Affiliations:** 1 School of Sport and Exercise Sciences, Liverpool John Moores University, Liverpool, UK; 2 Bioanalytical Facility, Norwich Medical School, University of East Anglia, Norwich, UK; 3 Clinical Biochemistry, Departments of Laboratory Medicine, Norfolk and Norwich University Hospital NHS Foundation Trust, Norwich, UK; 4 Departments of Diabetes and Endocrinology, Norfolk and Norwich University Hospital NHS Foundation Trust, Norwich, UK; 5 School of Health Sciences, Robert Gordon University, Aberdeen, UK; 6 Institute of Sport, Exercise and Health, University College London, London, UK

**Keywords:** strength training, vitamin C, connective tissue, glycine, proline, hydroxyproline

## Abstract

**Background:**

Resistance exercise (RE) stimulates collagen synthesis in skeletal muscle and tendon but there is limited and equivocal evidence regarding an effect of collagen supplementation and exercise on collagen synthesis. Furthermore, it is not known if a dose–response exists regarding the effect of hydrolyzed collagen (HC) ingestion and RE on collagen synthesis.

**Objective:**

To determine the HC dose–response effect on collagen synthesis after high-intensity RE in resistance-trained young men.

**Methods:**

Using a double-blind, randomized crossover design, 10 resistance-trained males (age: 26 ± 3 y; height: 1.77 ± 0.04 m; mass: 79.7 ± 7.0 kg) ingested 0 g, 15 g, or 30 g HC with 50 mg vitamin C 1 h before performing 4 sets’ barbell back squat RE at 10-repetition maximum load, after which they rested for 6 h. Blood samples were collected throughout each of the 3 interventions to analyze procollagen type Ⅰ N-terminal propeptide (PINP) and β-isomerized C-terminal telopeptide of type I collagen (β-CTX) concentration, and the concentration of 18 collagen amino acids.

**Results:**

The serum PINP concentration × time area under the curve (AUC) was greater for 30 g (267 ± 79 μg·L^−1^·h) than for 15 g (235 ± 70 μg·L^−1^·h, *P* = 0.013) and 0 g HC (219 ± 88 μg·L^−1^·h, *P* = 0.002) but there was no difference between 0 and 15 g HC (*P* = 0.225). The AUCs of glycine and proline were greater for 30 g than for 15 and 0 g HC (*P* < 0.05). Plasma β-CTX concentration decreased from −1 to +6 h (*P* < 0.05), with no differences between interventions.

**Conclusions:**

Ingesting 30 g HC before high-intensity RE augments whole-body collagen synthesis more than 15 g and 0 g HC in resistance-trained young males.

## Introduction

Musculoskeletal tissues, such as skeletal muscle and tendon, are crucial for generating and transmitting force to the bone, enabling movement. The structure and function of these tissues are therefore essential for musculoskeletal health and physical performance. Unfortunately, however, injuries to these tissues are common in athletes, with soft-tissue injuries making up the majority of all injuries in male athletes [1]. One of the biggest risk factors for soft-tissue injury is muscle weakness [2,3] and one of the most common methods used by athletes to mitigate this risk factor is to perform chronic resistance exercise (RE) [4]. Progressively overloading the muscle–tendon unit (MTU) in this way causes the muscle to adapt by hypertrophying and getting stronger [5], while the tendon also adapts by hypertrophying and increasing its stiffness and elastic modulus [6,7].

A stiffer tendon has a higher loading capacity, as there is a linear relationship between Young’s modulus and ultimate stress [8]. Given that collagen (mainly type I) makes up 60%–85% tendon dry weight [9], it is considered a crucial component in the tendon’s adaptation to RE, particularly as gains in tendon stiffness are thought to be influenced by both tendon hypertrophy and an increase in collagen fibril density [10,11]. An increase in type I collagen fibril content over time is likely the product of an overload-induced increase in collagen synthesis after each bout of RE.

Collagen synthesis can be assessed either directly from the overloaded tissue, for example, by measuring skeletal muscle or tendon collagen fractional synthetic rate (FSR), or indirectly from serum concentration of procollagen type Ⅰ C-terminal propeptide (PICP) or procollagen type Ⅰ N-terminal propeptide (PINP), which are both cleaved off during the maturation of procollagen to collagen. Indeed, an acute bout of RE in young males has been shown to increase patellar tendon collagen FSR [12] and serum PINP concentration [13]. This response is likely due to RE initiating mechanotransduction (that is, mechanical stress initiating fibroblast intracellular signaling) [14], and the secretion of growth factors [for example, transforming growth factor beta (TGF-β) and insulin-like growth factor-1 (IGF-I)], with these growth factors being crucial for procollagen formation in tendon [15,16]. Furthermore, these newly synthesized procollagen molecules undergo post-translational modifications, for which the presence of vitamin C is an essential co-factor during collagen synthesis [17], transport, and assembly into tendon [18].

Thus, RE appears crucial for inducing increases in serum PICP/PINP concentration and muscle–tendon collagen FSR, which may lead to changes in connective tissue properties in the longer term. Indeed, concomitant increases in serum PICP concentration and human Achilles tendon collagen content after two months' chronic RE, followed by an increase in Achilles tendon stiffness with a further month’s RE training [19], suggest that augmented tendon collagen synthesis and content are necessary to cause an increase in tendon stiffness. Not only is tendon stiffness important for mitigating soft tissue injury risk but it can also influence performance during “explosive” actions, as a stiffer tendon can transmit muscle force more effectively to the bone, thus increasing the rate of force development [20].

In addition to the role of exercise on collagen synthesis, ingestion of vitamin C-enriched collagen appears to further augment an exercise-induced increase in collagen synthesis in a dose–response manner, that is, 15 g gelatin increased serum PINP concentration by more than 2-fold compared with 5 g and 0 g gelatin [21]. This is currently the only study to investigate a dose–response effect of collagen ingestion on changes in collagen synthesis after exercise, albeit in jump-rope exercise not RE. Although no study has examined a collagen dose–response relationship without exercise, chronic collagen supplementation alone has been shown to induce improvements in bone mineral density [22] and cartilage health [23], suggesting that collagen ingestion might stimulate human connective tissue collagen synthesis independently of exercise. Thus, just as ingestion of 40 g whey protein has been shown to augment the muscle protein synthesis response to RE more than 20 g [24], it is possible that collagen ingestion may further augment the RE-induced rise in collagen synthesis [12,13] in a dose–response manner.

The aim of this study was therefore to investigate the effect of 30 g HC compared with 15 g and 0 g HC ingested before high-intensity back squat RE on whole-body collagen synthesis. We hypothesized that 30 g HC would elicit a greater serum PINP response than 15 g HC, which would induce a greater response than 0 g HC. We also hypothesized that 30 g HC would lead to greater blood availability of the amino acids necessary for collagen synthesis to occur, for example, glycine and proline.

## Methods

### Participants

**FIGURE 1:**
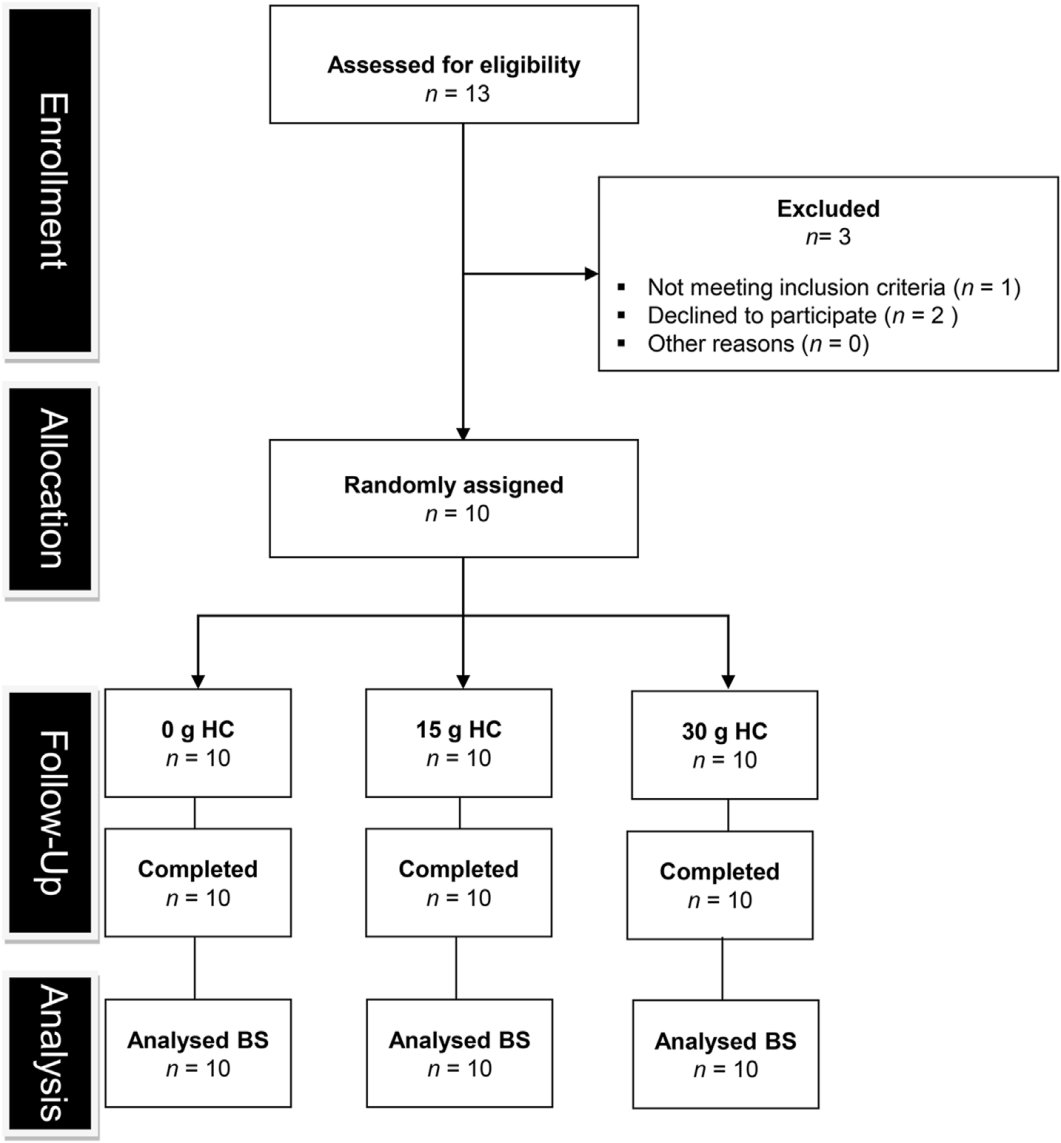
CONSORT flow diagram. HC, hydrolyzed collagen; BS, blood samples collected for serum procollagen type I N-terminal propeptide (PINP) concentration, plasma β-isomerized C-terminal telopeptide of type I collagen (β-CTX) concentration, and serum amino acid concentration.

Thirteen healthy young males volunteered to take part in the study. However, 3 were excluded before participation because of not meeting the inclusion criteria (*n* = 1) and declining to proceed with participation (*n* = 2) (Figure 1). Therefore, 10 resistance-trained, healthy young males (mean ± SD; age: 26 ± 3 y, height: 1.77 ± 0.04 m, body mass: 79.7 ± 7.0 kg, 4 ± 3 y of RE experience), who performed RE 4 ± 1 times per week, provided written informed consent before completing this study. The study was registered at https://clinicaltrials.gov/ (identifier: NCT05932771), was approved by Liverpool John Moores University Ethics Committee (approval number: 18/SPS/059) and complied with the Declaration of Helsinki. Participants were recruited from a university student population, and recruitment began in January 2019 and data collection was completed in August 2019. To be eligible to participate, volunteers had to be male, have at least 12 months' resistance training experience (including barbell back squat exercise performed at least once a week), and to be free from musculoskeletal injury. Volunteers were excluded if they had a history of patellar tendon pathology, were vegan (because of the bovine source of HC), consumed nutritional supplements or medication purported to have beneficial effects on muscle–tendon properties (for example, antioxidants, protein, etc.), had sustained a lower-limb injury in the previous 6 months, smoked, or were <18 or >30 y old.

### Experimental design

**FIGURE 2:**
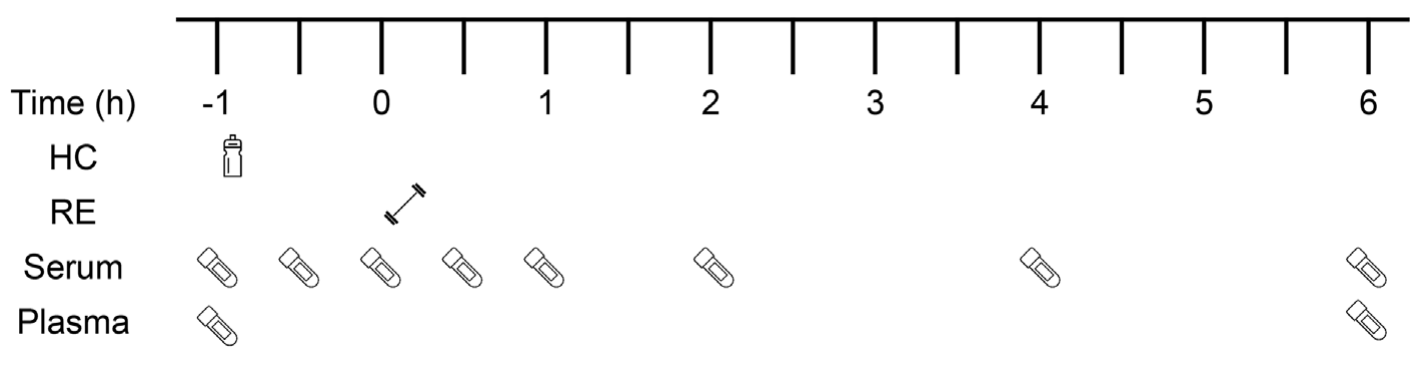
Schematic diagram of the experimental protocol. HC, hydrolyzed collagen; RE, resistance exercise; *−*1 h, fasted blood sample followed immediately by HC consumption; ; 0 h, start of RE; Blood serum collected at eight time points throughout each intervention; blood plasma collected at two time points (-1 h and 6 h post-RE).

This study was a double-blind, randomized crossover design. All participants attended the laboratory on 4 occasions, each separated by a week, and no strenuous physical activity was performed 48 h before each visit. Visit 1 was used to assess barbell back squat 10 repetition maximum (10-RM), whereas visits 2–4 began with participants consuming a drink containing 0 g, 15 g, or 30 g HC (each containing 50 mg vitamin C), followed by 4 sets of 10-RM barbell back squat RE (with 2 min rest in between sets), which typically took 20 min to complete. The 3 interventions (with a 7-d wash-out period between each intervention) were performed at the same time of day (08:00–15:00), after a 10-h overnight fast. After consuming the supplement and completing the RE, participants rested for 6 h and 10 × 5-mL blood samples were collected at different times points over a 7-h period (Figure 2). In addition to the supplement, only water was allowed to be consumed (ad libitum) during each intervention. Participants were instructed to record their dietary intake on the day before their first intervention and to replicate that dietary behavior on the day preceding each of the subsequent interventions.

### The barbell back squat 10-RM assessment and 10-RM bout during each intervention

**FIGURE 3:**
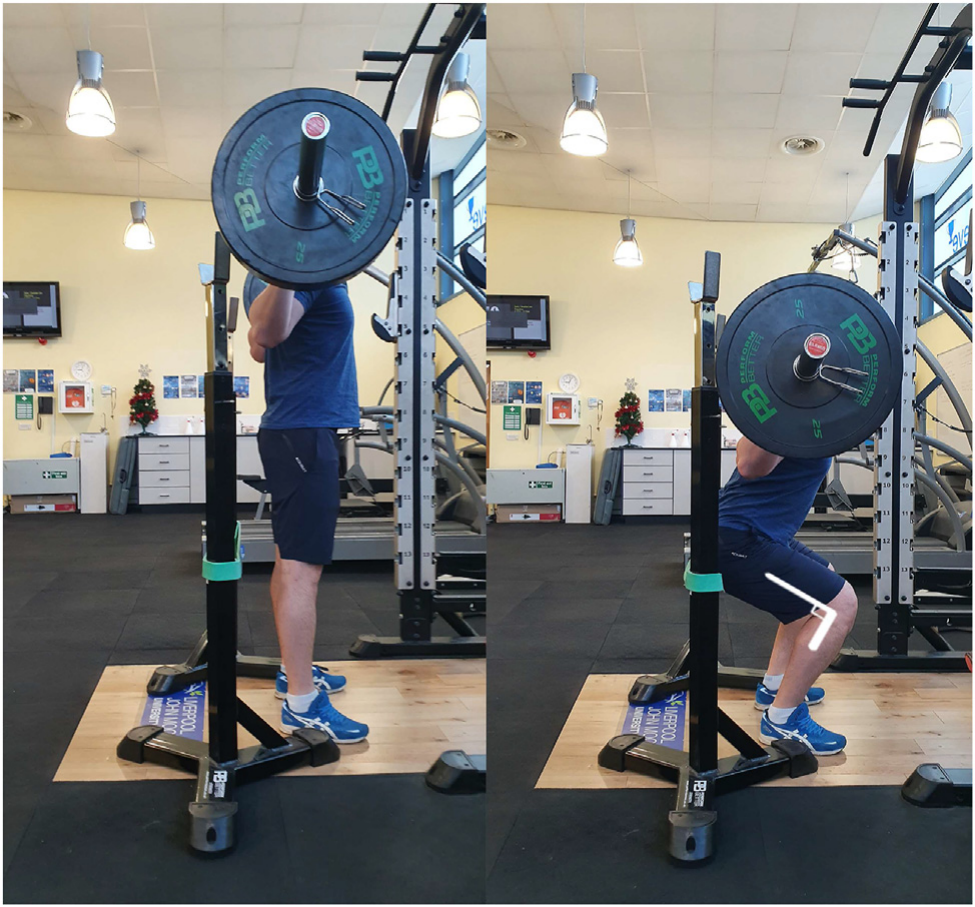
90° barbell back squat. A resistance band was attached to the squat rack to indicate when the participant had reached 90° knee flexion during each repetition of the back squat 10-RM.

The squat depth during the barbell back squat was standardized for all participants to induce the same mechanical loading on the quadriceps femoris MTU during all 3 experimental interventions. Participants were instructed to place a 20 kg Olympic barbell on their shoulders (the high bar position), place their feet shoulder-width apart (foot location was marked on the floor for subsequent sets) and descend until their knee joint angle reached 90°, measured using a goniometer. While participants held the position at 90° knee flexion, the vertical distance from the floor to the ischial tuberosity was measured. The 10-RM assessment was performed in a squat rack and a resistance band was stretched across both sides of the squat rack to indicate the participant’s 90° depth (Figure 3). A warm-up comprised 2 dynamic exercises (low lunge and squat to stand) before the actual 10-RM assessment, which comprised the following sets of barbell back squat: 10 repetitions with the 20 kg barbell, 8 repetitions at 50% of the estimated 10-RM, 4 repetitions at 70%, and 1 repetition at 90% of the estimated 10-RM). After a 5-min rest period, participants performed 10-RM attempts separated by 5-min rest periods until 10-RM load was obtained. Two researchers observed each test procedure to provide a cue when the participant’s proximal hamstrings/gluteus maximus touched the elastic band and to spot the participant. The 10-RM bout during each experimental intervention was preceded by a similar warm-up, that is, 2 dynamic exercises followed by 10 repetitions’ barbell back squat with the 20 kg barbell, 8 repetitions at 50% of the measured 10-RM, 4 repetitions at 70% 10-RM, and 1 repetition at 90% 10-RM. The barbell back squat 10-RM load was 118 ± 21 kg during all 3 interventions.

### Nutritional supplementation

Before commencing each intervention, a laboratory technician (independent to the study) made up the supplement and randomized the order of HC dose (Excel 2016, Microsoft) for each participant. For each intervention, the technician recorded the date, randomly allocated intervention number (1, 2, or 3), and the corresponding HC dose. The study investigators and participants were blinded to HC dose until after all analyses were completed, after which time the technician provided the lead researcher with the participants’ intervention numbers and corresponding HC doses. Three doses of HC (0 g, 15 g, and 30 g, Myprotein) with 50 mg vitamin C powder (Holland and Barrett Retail Limited) were dissolved in 300 mL water in an opaque drinks bottle. To match the calories of 30 g HC in the other 2 interventions, 34.1 g and 15.4 g maltodextrin (Myprotein) was used in the 0 g and 15 g HC interventions, respectively. Although the supplements were described by the manufacturers as “flavorless,” 4 g non-caloric sweetener (Truvia®, SilverSpoon) was added in all drinks to mask any potential taste difference between interventions. The amino acid profile of the HC supplement is shown in Table 1.

**TABLE 1:** Amino acid composition of the hydrolyzed collagen supplement

Amino acids	Weight (%)
Glycine	21.0
Proline	12.8
Hydroxyproline	12.2
Glutamic acid	10.3
Alanine	8.9
Arginine	7.3
Aspartic acid	6.0
Lysine	3.5
Serine	3.1
Leucine	2.7
Valine	2.4
Phenylalanine	2.1
Threonine	1.9
Hydroxylysine	1.5
Isoleucine	1.5
Histidine	1.1
Tyrosine	1.0
Methionine	0.9

### Blood sampling

The BD Nexibia^TM^ closed IV catheter system (22 G, Becton, Dickinson and Company) was inserted into a peripheral vein in the right antecubital fossa by a trained phlebotomist. A dressing band (3M™ Tegaderm™ I.V. Advanced Securement Dressing, 3M Health Care) then covered the catheter to secure the catheter site and to keep it clean. Eight 5-mL venous blood samples were collected in specialized serum collection tubes [BD Vacutainer™ Serum Separation Tube (SST™) II Advance, Dickinson and Company] at the following time points: at rest immediately before HC ingestion, 0.5 h post-HC ingestion, 1 h post-HC ingestion, 0.5 h post-RE, 1 h post-RE, 2 h post-RE, 4 h post-RE, and 6 h post-RE for serum preparation (Figure 2). The samples were used to analyze serum PINP and amino acid concentration. Two × 5 mL venous blood samples were collected in EDTA plasma collection tubes [BD Vacutainer™ Hemogard Closure Plastic K2-EDTA Tubes, Dickinson and Company] at rest immediately before HC ingestion and 6 h post-RE for plasma preparation. These samples were used to analyze plasma β-isomerized C-terminal telopeptide of type I collagen (β-CTX). The catheter was flushed by 3 mL sterile prefilled flush syringes containing sodium chloride 0.9% (BD PosiFlush™ Pre Filled Saline Syringe, Dickinson and Company) every 30 min to clean and prevent blood from clotting and blocking the catheter. The SSTs were stored in a tube rack for 30 min for clotting at room temperature and the EDTA tubes were immediately placed on ice before being centrifuged at 1000 × g at 4°C for 10 min. The serum and plasma samples were then aliquoted into 5 mL round-bottom polystyrene tubes (Falcon™, Thermo Fisher Scientific) and stored at −80°C until subsequent analysis.

### Blood analyses

Markers of collagen synthesis and breakdown were analyzed by measuring the circulating concentration of PINP and β-CTX, respectively. Furthermore, circulating collagen amino acid concentrations were measured throughout the entirety of each intervention. PINP analyses were performed at Liverpool John Moores University, whereas β-CTX and amino acid profile analyses were performed at the Bioanalytical Facility, University of East Anglia.

#### PINP

Six serum samples (at rest before HC ingestion, 0.5 h post-RE, 1 h post-RE, 2 h post-RE, 4 h post-RE, and 6 h post-RE) were used to measure serum PINP concentrations using an ELISA according to the manufacturer’s instructions (USCN Life Sciences). The intra-assay coefficient of variation (CV) was <10% and the inter-assay CV was <12%, with a detection range of 2.47–200 μg·L^−1^, and sensitivity of <0.91 μg·L^−1^. The ELISA absorbance readings were performed at 450 nm, using a Clariostar microplate reader (BMG Labtech). The concentration × time total AUC for PINP and amino acids (see below) were calculated using Prism software (version 9.4.1, GraphPad Inc.).

#### β-CTX

EDTA plasma concentrations of β-CTX were measured using electrochemiluminescence immunoassay on a Cobas e601 analyzer (Roche Diagnostics). The inter-assay CV for β-CTX was ≤3% between 0.2 and 1.5 μg·L^−1^ with the sensitivity of 0.01 μg·L^−1^.

#### Amino acid profile

Eight serum samples (at rest immediately before HC ingestion, 0.5 h post-HC ingestion, 1 h post-HC ingestion, 0.5 h post-RE, 1 h post-RE, 2 h post-RE, 4 h post-RE, and 6 h post-RE) were used to assess the concentration of 18 amino acids associated with collagen composition (glycine, proline, hydroxyproline, glutamic acid, alanine, arginine, aspartic acid, lysine, serine, leucine, valine, phenylalanine, threonine, isoleucine, histidine, tyrosine, methionine, and glutamine, but not hydroxylysine). All 18 amino acid concentrations were measured simultaneously using an anionic ion-pair reverse phase liquid chromatography with tandem mass spectrometry (LC-MS/MS) system after derivatization of the amino acid with *n*-butanol hydrogen chloride. The LC-MS/MS system used a Micromass® Quattro Ultima™ Pt coupled to an Agilent 1100 series high-performance liquid chromatography binary pump. Electrospray ionization source operating in positive ion mode, mass detection for each amino acid butyl ester was achieved in multiple reaction monitoring mode. Certified amino acid standards were purchased from Wacko Chemicals GmbH and Sigma–Aldrich. Internal standards used were glucosaminic acid and S-(2-Aminoethyl)-L-cysteine hydrochloride (Sigma–Aldrich) and L-Citrulline-2,3,3,4,4,5,5-d7 (Isoscience). Three internal quality controls (QC) at low, medium and high concentrations were made from pooled human serum. For each batch of analysis, 10 μL of standards, QC, and test samples were added to a microcentrifuge tubes, to which 440 μL of internal standards made up in 0.1M hydrochloride in methanol was added. The mixture was vortexed twice, each time allowed to stand for 10 min, then centrifuged at 10,800 × g for 5 mins. The supernatant was then transferred into a borosilicate tube and dried to completeness under nitrogen gas at a temperature of 60°C. 100 μL of 3N *n*-butanol hydrogen chloride was added to the dried residue, vortex mixed, capped, and incubated at 60°C for 7 min. After butylation, the mixture was dried completely under nitrogen gas, and then reconstituted with 250 μL of 12% acetonitrile:water containing 0.025% heptafluorobutyric acid (HFBA). After a final vortex mix, the samples were transferred to a polypropylene autosampler vial for injection into the LC-MS/MS.

Chromatographic separation was achieved using a Modus AAC 100 × 2.1 mm 3 μm column (Chromatography Direct Ltd) maintained at 40°C. Anionic ion-pair reagent HFBA was added to the mobile phases to improve analyte interaction with the stationary phase. A gradient elution profile at a flow rate of 350 μL·min^−1^ was used throughout. Initial conditions were 88% mobile phase A (0.025% HFBA in water) and 12% mobile phase B (0.025% HFBA in acetonitrile). This was held for 30 s. Mobile phase B was increased linearly to 20% at 10 min, with a further linear increase in mobile phased B to 60% at 15 min. This was held constant until 16.9 min and returned to the initial conditions at 17 min. Injection volume was 10 μL with an injection cycle time of 20 min. The assay range was 0–2000 μmol·L^−1^ for all 18 amino acids studied. Inter-assay precision CV for all amino acids was between 3.3% and 10.3%.

### Statistical analyses

Data were analyzed using the statistical software package SPSS (Version 26, IBM Inc.). The sample size was estimated before conducting the study with G∗Power software (version 3.1.9.6, Heinrich-Heine-Universität Düsseldorf). The estimation was performed using a large effect size (*η*_p_^2^ = 0.22), on the basis of the results from Shaw et al. [21], which demonstrated a 2-fold increase in the serum PINP concentration × time AUC after exercise with 15 g compared to 5 g gelatin ingestion. The results from our a priori power calculation deemed a minimum of 8 participants was necessary to detect an effect of HC dose [1-way repeated measures analysis of variance (ANOVA); α: 0.05; power: 0.80]. We recruited 10 participants to account for an expected 10–20% drop out. Using the Shapiro–Wilk test, all data were deemed to be normally distributed except for the amino acid data. The latter data were therefore log transformed before undergoing subsequent statistical analyses. One-way within-subject ANOVA models were performed to compare baseline (−1 h) concentrations of PINP and β-CTX in all 3 trails. Two-way within-subject ANOVAs (dose × time) were performed to detect changes in serum PINP and amino acid concentrations over time. To detect changes in plasma β-CTX concentration, a two-way within-subject ANOVA (dose × time) was performed. One-way repeated measures ANOVA models were performed to detect dose-dependent differences in concentration × time AUCs for PINP and each of the 18 amino acids analyzed. Where Mauchly’s test of sphericity had been violated, Greenhouse–Geisser (ε < 0.75) or Huynh–Feldt (ε > 0.75) corrections were applied. Where a main effect of HC dose existed, Fisher’s LSD post-hoc pairwise comparisons were performed to reveal which doses differed. Partial eta squared (*η*_p_^2^) effect sizes were reported for each statistical model, and the thresholds for *η*_p_^2^ are defined as small (*η*_p_^2^ = 0.01), medium (*η*_p_^2^ = 0.06), and large (*η*_p_^2^ = 0.14) [25]. All data analyses matched the research design, as there were no missing data for any of the dependent variables. The level of statistical significance was set at *P* < 0.05 and all data are presented as mean ± standard deviations with 95% confidence intervals (CI, where applicable), unless stated otherwise.

## Results

### Serum PINP concentration and AUC

**FIGURE 4:**
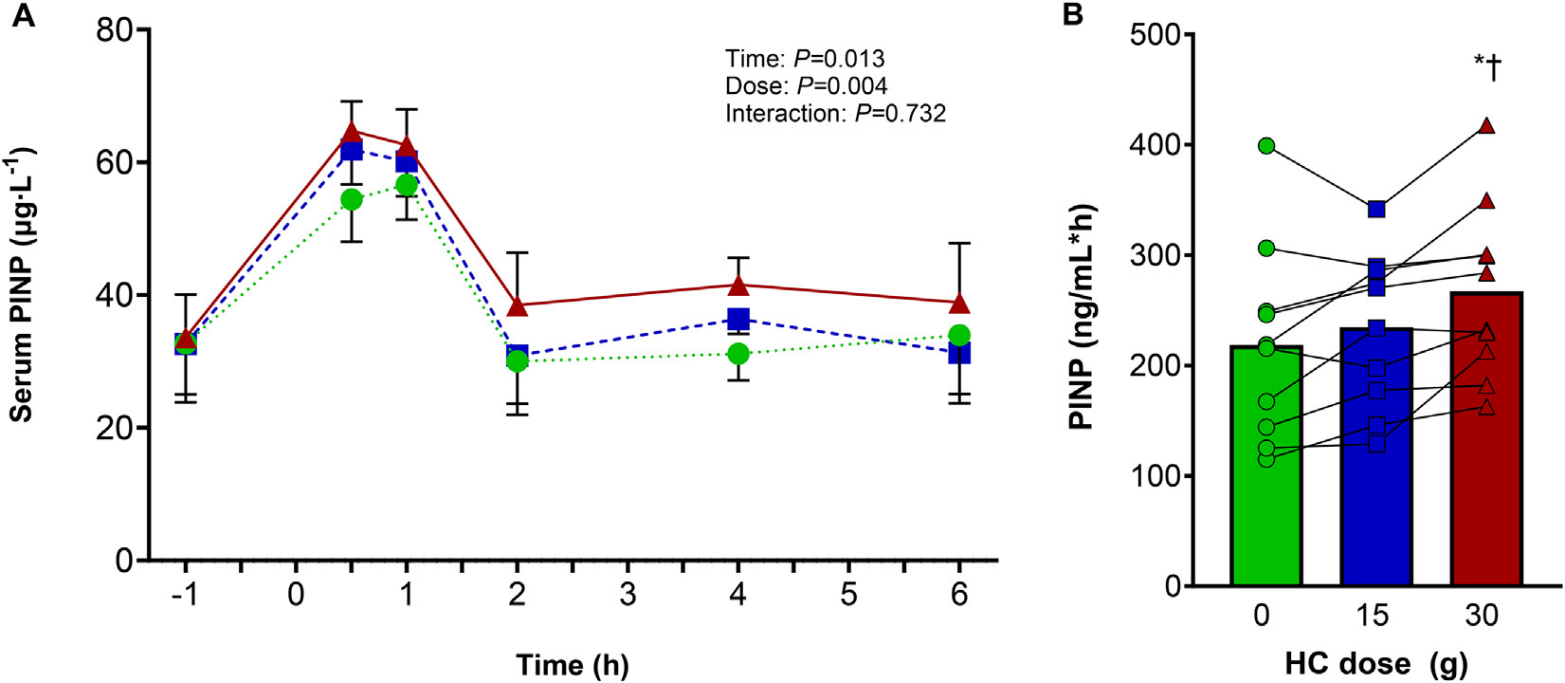
Collagen synthesis after hydrolyzed collagen (HC) ingestion (−1 h) and performing resistance exercise (0 h). (A) Serum PINP concentration and (B) serum PINP concentration × time AUC after 0 g HC (green circles), 15 g HC (blue squares), and 30 g HC (red triangles) ingestion. ∗Greater than 0 g HC (*P* = 0.002); ^†^Greater than 15 g HC (*P* = 0.013). Values represent mean ± SEM.

Baseline serum PINP concentrations for the 0 g, 15 g, and 30 g HC interventions did not differ [*P* = 0.990, η_p_^2^ = 0.001; 0 g HC 32.7 ± 28.0 (95% CI: 12.7, 52.7) μg·L^−1^; 15 g HC 32.7 ± 24.2 (95% CI: 15.4, 49.9) μg·L^−1^; 30 g HC 32.3 ± 21.6 (95% CI: 16.9, 47.7) μg·L^−1^]. Regarding serum PINP concentration, there was a main effect of HC dose (*P* = 0.004, η_p_^2^ = 0.462) and time (*P* = 0.013, η_p_^2^ = 0.458) but no dose × time interaction effect (*P* = 0.732, η_p_^2^ = 0.071, Figure 4A). These results suggest that the dose effect was not time specific. Post-hoc pairwise comparisons revealed that 30 g HC had a greater PINP response than 0 g HC (*P* = 0.002) and 15 g HC (*P* = 0.020), whereas 15 g HC did not differ from 0 g HC (*P* = 0.245). Regarding the serum PINP concentration × time AUC, there was a main effect of HC dose (*P* = 0.001, η_p_^2^ = 0.517), and post-hoc pairwise comparisons revealed that 30 g HC had a greater AUC [267 ± 79 (95% CI: 211, 323) μg·L^−1^·h] than 15 g HC [235 ± 70 (95% CI: 184, 284) μg·L^−1^·h, *P =* 0.013], and 0 g HC [219 ± 88 (95% CI: 155, 281) μg·L^−1^·h, *P =* 0.002], but 15 g HC AUC did not differ from 0 g HC AUC (*P* = 0.225, Figure 4B).

### Plasma β-CTX

**FIGURE 5:**
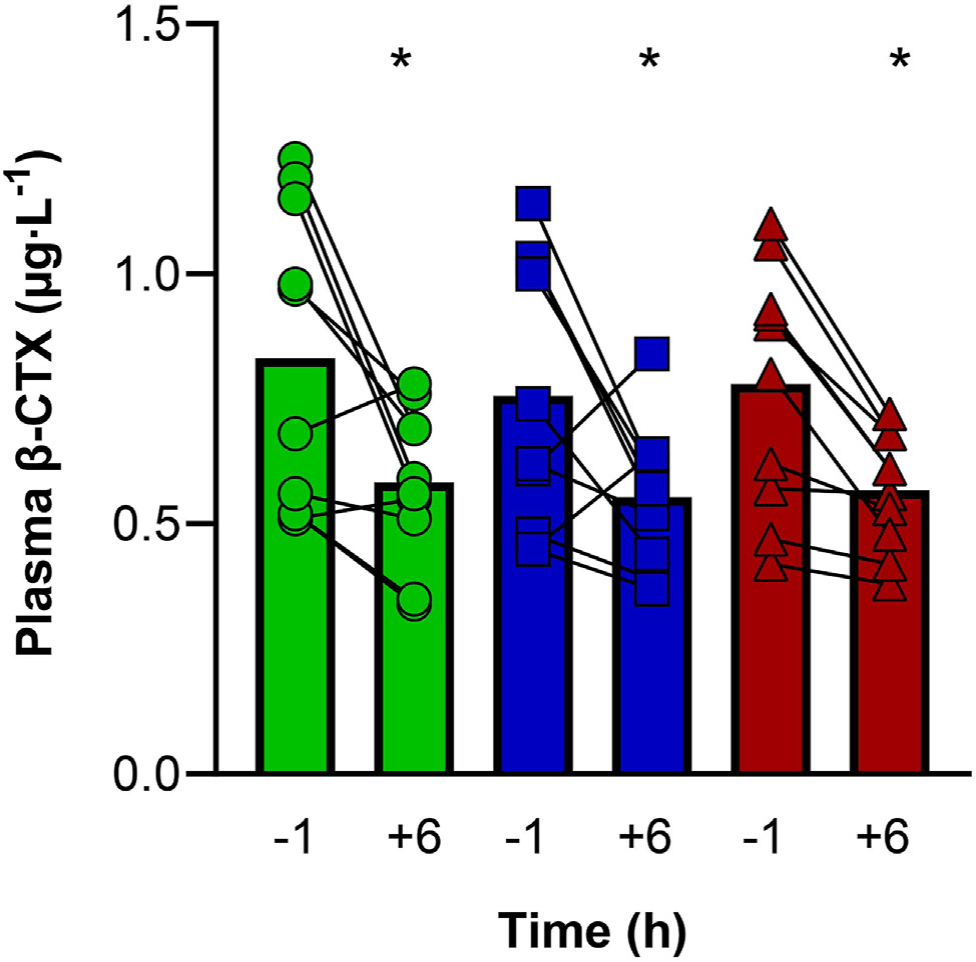
Collagen breakdown after hydrolyzed collagen (HC) intake and performing resistance exercise. Plasma CTX-1 concentrations after 0 g HC (green circles), 15 g HC (blue squares), and 30 g HC (red triangles) ingestion. ∗Lower than *−*1 h (*P* < 0.05).

Baseline plasma β-CTX concentrations for the 0 g, 15 g, and 30 g HC interventions did not differ [*P* = 0.311, η_p_^2^ = 0.122; 0 g HC 0.8 ± 0.3 (95% CI: 0.6, 1.0) μg·L^−1^; 15 g HC 0.8 ± 0.3 (95% CI: 0.6, 0.9) μg·L^−1^; 30 g HC 0.8 ± 0.2 (95% CI: 0.6, 0.9) μg·L^−1^]. There was a main effect of time (*P* = 0.007, η_p_^2^ = 0.577) but no main effect of HC dose (*P* = 0.286, η_p_^2^ = 0.127) and no dose × time interaction (*P* = 0.748, η_p_^2^ = 0.031), that is, plasma β-CTX concentration decreased from −1 h (before HC ingestion and RE) to 6 h post-RE for all 3 interventions, with no difference between interventions (Figure 5).

### Serum amino acid concentrations

**FIGURE 6:**
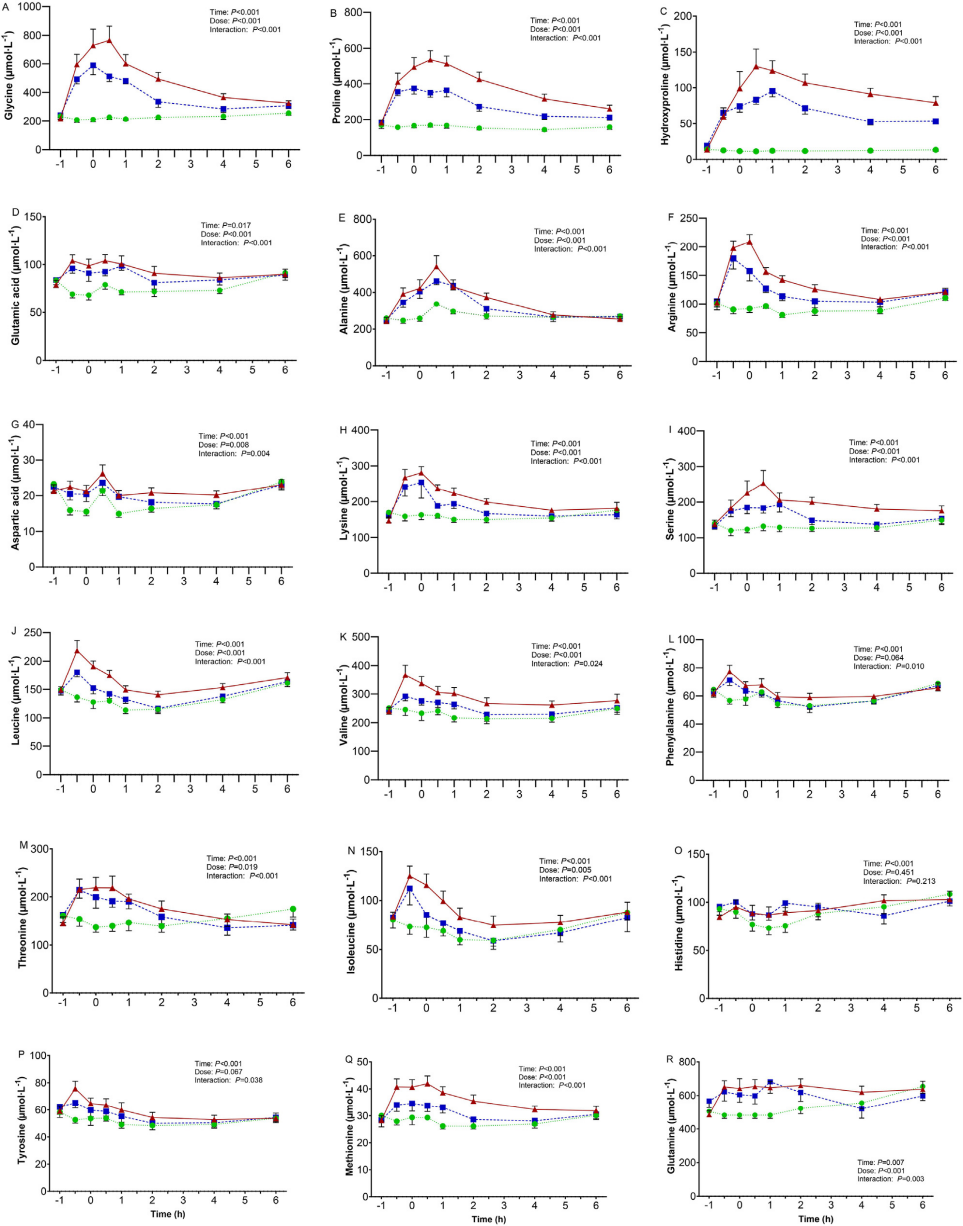
Concentrations of 18 serum amino acid at before ingesting hydrolyzed collagen (−1 h), 1 h after ingesting 0 g (green circles), 15 g (blue squares), or 30 g (red triangles) (HC) (+1 h) and then performing barbell back squat exercise at time point 0 h. Values represent mean ± SEM.

Serum concentrations over the 7-h period of each intervention of the 18 amino acids that constitute type I collagen are shown in Figure 6. The main effects of time, dose, and dose × time interaction effects for each amino acid are denoted in Figure 6. There were main effects of dose for 14 amino acids (glycine, proline, hydroxyproline, glutamic acid, alanine, arginine, aspartic acid, lysine, serine, leucine, valine, isoleucine, methionine, and glutamine), with 30 g HC demonstrating higher serum concentrations than 0 g. Of those amino acids, glycine, proline, hydroxyproline, arginine, lysine, serine, leucine, valine, isoleucine, and methionine in 30 g HC showed higher serum concentrations than 15 g HC. All amino acids except for histidine (*P* > 0.05) showed a dose × time interaction effect, and there was no main effect of dose for histidine (*P* = 0.451).

## Discussion

This study is the first to investigate the effect of high-intensity RE and 0 g, 15 g, and 30 g HC supplementation on whole-body collagen turnover in an homogenous group of resistance-trained, healthy, young men. We found that the serum PINP concentration × time AUC for the 30 g HC intervention was greater than for the 15 g and 0 g HC interventions. Furthermore, these results were consistent with greater increases in the appearance of key amino acid constituents of collagen (for example, glycine and proline) within the blood after ingestion of 30 g HC versus 15 g and 0 g. Therefore, at least 30 g HC is required to provide greater exogenous collagen amino acid availability, which appears to be a key factor for optimizing collagen synthesis after high-intensity RE in resistance-trained, young males.

To address the aims of our study, we measured serum PINP after RE with different doses of HC. We chose high-intensity back squat RE to target the quadriceps MTU, because the human patellar tendon appears to hypertrophy only after prolonged periods of high-intensity [6,7] and not moderate-intensity [26] resistance training. In this study, the 7-h experimental design was based on a significant increase in muscle and tendon collagen FSR at 6 h post-exercise after a single RE session in healthy young males [12]. Although the significantly elevated muscle collagen FSR was similar at 6 h post- and 24 h post-RE [12], tendon collagen FSR appeared to be further augmented at 24 h post- compared with 6 h post-RE, although it is not stipulated in the article whether tendon collagen FSR measured at these 2 time points differed significantly. In a separate study, serum PICP concentration was significantly higher 48 h post-exercise after 50 maximal concentric knee extensions in healthy young males [27]. Thus, it is possible that, had each of our three interventions lasted 24–48 h post-RE, we may have observed further increases in serum PINP concentration, and possibly a larger effect of HC ingestion on these increases.

The 30 min high-intensity RE model we employed in our study was associated with peak serum PINP concentrations of ∼60 μg·L^−1^ (regardless of dose), which occurred 30–60 min after the onset of RE (and 90–120 min after supplement ingestion). We chose to measure serum PINP concentration because it is a reliable biomarker of collagen synthesis, being a procollagen peptide that is cleaved off during maturation from procollagen to collagen [28]. The similar PINP concentration at +0.5 and +1 h post-RE for all 3 doses (including 0 g HC) suggests the increase in PINP concentration observed within the first hour after starting the RE occurred as a consequence of the RE, rather than HC ingestion. However, it is possible that our data at +0.5 h post-RE may have been influenced by an increase in blood flow. A distinction should be made between flux, that is, the total amount of PINP passing through the blood registered at any given time, and concentration, that is, the ratio of PINP to the volume of serum. Blood flow rises 20-fold and 7-fold in the calf muscle and peritendinous area of the Achilles tendon, respectively, during repeated plantar flexion contractions in healthy individuals [29]. However, an increase in blood flow to the peritendinous tendon has been shown to return to resting levels within a few minutes of finishing the same type of exercise [30]. Therefore, the fact that serum PINP concentration was still at its peak at +1 h in the current study, that is, 30 min after RE ended, when cardiac output would be expected to have returned to resting rates after lower-limb RE [31], it is likely that this increase in PINP concentration was because of an increase in RE-induced collagen synthesis, rather than blood flow. Serum PINP concentration subsequently decreased to baseline values in the 0 g and 15 g interventions, and remained at this level for the rest of those interventions, whereas it decreased but remained more elevated in the 30 g intervention, which resulted in the higher AUC in the 30 g HC intervention compared with the 0 g and 15 g HC interventions.

In contrast to our results, Aussieker et al. [32] recently found that 30 g HC ingestion with 6 sets of 8–15 repetitions at 60% estimated 1-RM barbell back squat did not augment vastus lateralis muscle connective tissue protein FSR or circulating PINP concentration more than RE with 30 g whey protein ingestion or RE alone in different groups of young males and females. A number of differences in study design may help explain this discrepancy between studies. First, the between-group design used by Aussieker et al. [32] may have introduced more between intervention variability (thus potentially confounding an effect of HC) compared to a within-group crossover design, as used in the present study. Second, estrogen is known to affect skeletal muscle and tendon collagen synthesis in females [33,34], and the use of a mixed-sex cohort by Aussieker et al. [32], rather than a 100% male cohort as used in the present study, may have increased within-group variability in connective tissue protein FSR. Third, vitamin C was not consumed during the interventions by Aussieker et al. [32], which began after an overnight fast. As vitamin C is required for the biosynthesis of collagen [17] and humans are unable to store it in the body or synthesize it endogenously [35], this may have limited muscle connective tissue protein FSR. Finally, it should be noted that Aussieker et al. [32] measured connective tissue protein FSR in skeletal muscle and not tendon. Tendon has a 60–85% type I collagen content [9] compared with just ∼5% in skeletal muscle [36]. Therefore, a beneficial effect of HC supplementation on collagen FSR is more likely to be seen in tendon than skeletal muscle.

Contrary to Aussieker et al. [32] and in accordance with our findings, Shaw et al. [21] found that serum PINP concentration was greater after jump-rope exercise with gelatin supplementation in a dose–response manner. Because of different time points used to measure serum PINP concentration, a direct comparison of peak concentration between studies is not possible. Nevertheless, the serum PINP AUC was greater in our 30 g HC intervention compared with our 15 g and 0 g interventions (with no difference between our 0 g and 15 g interventions, Figure 4), whereas Shaw et al. [21] found a greater effect of 15 g compared with 5 g and 0 g gelatin. This suggests that the different exercise models used may require different doses of exogenous collagen to optimize the collagen synthetic response for that particular exercise.

Regarding the amino acids that constitute collagen (for example, glycine, proline, hydroxyproline, etc.), these peaked in circulation around 1–1.5 h after ingestion of 30 g HC in our study (Figure 6). This was in line with previous studies, which involved the ingestion of 15 g gelatin or 20 g or 30 g collagen peptides in healthy young populations [21,32,37]. This similarity between studies indicates that the maximal rate of amino acid absorption occurs approximately an hour after ingestion of collagen in healthy young males, regardless of the dose and type of collagen supplementation. We also observed that the average concentrations of glycine, proline, hydroxyproline, arginine, lysine, serine, leucine, valine, isoleucine, and methionine were greater after ingestion of 30 g HC compared with 15 g and 0 g HC. The high availability of collagen amino acids in the 30 g HC intervention might have promoted a greater collagen synthetic response in two ways. First, the greater abundance of key amino acids may have simply provided more of the essential components to increase collagenous tissue content after an overload-induced stimulation of collagen synthesis in the lower-limb MTUs. Second, they may have stimulated mammalian target of rapamycin complex 1 (mTORC1) phosphorylation independently of muscle contraction/stretch-activated mechanisms, in a similar manner to amino acid stimulation of skeletal muscle myofibrillar protein synthesis via mTORC1 activation [38]. For example, after treating chondrocytes from bovine cartilage with 1.5 mM glycine, proline, or lysine for 15 d, type II collagen synthesis was 1.6 times greater after glycine treatment compared with proline treatment and 2 times greater compared with lysine treatment [39]. Furthermore, human skin fibroblasts treated with 5 mM proline for 48 h demonstrated an increase in collagen type I α1 expression [40], whereas proline and hydroxyproline also increased TGF-β expression in human fibroblasts [41], which would be expected to cause phosphorylation of protein kinase B (Akt) and mTORC1 (thus explaining the increase in collagen synthesis and gene expression). Considering the above mechanisms and that collagen synthesis is stimulated via phosphorylation of Akt and mTORC1 in response to mechanical loading in cultured human tendo.n-derived stromal cells [42], and that mechanical loading increases in TGF-β expression and type I collagen expression in rat Achilles tendon [43], independent RE- and amino acid-associated signaling pathways likely explain our findings that high-intensity RE with 30 g HC supplementation stimulates collagen synthesis more than RE with 15 g HC ingestion or RE alone.

Concerning collagen breakdown, β-CTX is released into circulation from mature type I collagen during degradation, and therefore serves as a reliable biomarker of collagen breakdown [44]. In this study, regardless of HC dose, plasma β-CTX concentration decreased by ∼30% from −1 to 6 h post-RE (Figure 5). This may have been caused by the high-intensity RE stimulating collagen synthesis (Figure 4), subsequently inhibiting collagen breakdown. This hypothesis is supported by currently unpublished data from our laboratory in middle-aged males, suggesting the decrease in circulating β-CTX concentration occurs immediately after RE and remains lower for the subsequent 6 h of rest, regardless of the HC dose. Alternatively, the reduction may have been associated with circadian rhythm, as Qvist et al. [45] reported that resting serum β-CTX concentrations in males and pre- and postmenopausal females (aged 24–73 y) peaked at 08:00, then sharply decreased between 11:00 and 14:00. Nevertheless, as tissue turnover is determined by the rates of both synthesis and breakdown, the role of collagen degradation on the regulation of collagen turnover is crucial. For example, avian skeletal muscle exposed to mechanical loading led to an increase in collagen synthesis, which was accompanied by a decrease in the degradation of newly synthesized collagen, as well as an increase in the degradation of mature collagen [46]. Thus, further research is necessary to elucidate the effects of RE and HC supplementation on degradation of newly synthesized and mature collagen for the regulation of collagen turnover.

A limitation of this study is that our assessment of collagen synthesis was indirect, that is, we measured serum PINP concentration rather than harvesting tendon biopsies, with which we could have assessed tendon PINP concentration or collagen FSR directly. However, human tendon [47] and serum PINP concentration [13] increases after an acute bout of exercise, and serum PINP can remain elevated for up to 4 days after resistance-type exercise in healthy young males [27]. Nevertheless, future studies should investigate the effect of HC ingestion with RE on connective tissue collagen synthesis by measuring circulating and tendon PINP concentration, and tendon collagen FSR simultaneously.

In conclusion, we have demonstrated for the first time that a single bout of high-intensity, lower-limb RE with 30 g HC ingestion increased whole-body collagen synthesis more than RE with 15 g or 0 g HC in resistance-trained young males. This higher response was likely related to the greater availability of key amino acids after the ingestion of 30 g HC compared with 15 g and 0 g HC. This may have implications for augmenting tendon adaptation to high-intensity resistance training when 30 g HC is ingested in combination with RE over a prolonged period of time. Future studies should also investigate if a dose–response exists regarding high-intensity RE with HC supplementation in resistance-trained young females.

## Author contributions

The authors’ responsibilities were as follows – RME: study conception and design; JL: data collection; JL: data curation; JL, RME, JCYT, RD, JD: data analysis; RME, DRC, CES: supervision; JL: writing—original draft; RME, JCYT, RD, JD, WDF, KE, DRC, CES: writing—review, editing, and approval of final draft.

## Conflict of interest

The authors declare that the research was conducted in the absence of any commercial or financial relationships that could be construed as a potential conflict of interest.

## Funding

The authors reported no funding received for this study.

## Data availability

Data described in the article, code book, and analytic code will be made available upon request from the corresponding author pending application and approval.

## Appendix A Supplementary data

Multimedia component1 Multimedia component1
