# Supplementary material for: The Collagen Synthesis Response to an Acute Bout of Resistance Exercise Is Greater when Ingesting 30 g Hydrolyzed Collagen Compared with 15 g and 0 g in Resistance-Trained Young Men
Source: J Nutr. 2023 Nov 24;154(7):2076–86. doi: 10.1016/j.tjnut.2023.10.030 (PMC11282471; doi:10.1016/j.tjnut.2023.10.030)
Supplement: Multimedia component1 [file mmc1.docx]

**Table 1**. Checklist of information to include when reporting research in sport nutrition and exercise metabolism. This table was adopted from Betts et al. (2020)

| Section | Item | Checklist | Page (pp.)/line number (LN) |
| --- | --- | --- | --- |
| **Title** | 1a  1b | State the independent (groups/conditions) and dependent (outcome) variables  Identify the study population or case | pp.1  pp.1 |
| **Abstract** | 2a  2b  2c | Specify the research design, methods, and characteristics of study population  Report a balanced account of the results and cite actual data  Restrict conclusions to measured variables, without speculation or unsupported recommendations | LN 8 – 14  LN 15 – 19  LN 20 – 21 |
| **Introduction** | 3a  3b | Present a scientific rationale based on an objective review of available evidence  State the aims, objectives, research questions, and/or hypotheses | LN 25 – 84  LN 84 – 89 |
| **Methods**  *Ethics*  *Design*  *Sampling*  *Interventions*  *Measurement*  *Randomization*  *Blinding*  *Standardization*  *Order effects*  *Statistics* | 4  5  6a  6b  6c  6d  7  8a  8b  8c  9  10  11  12  13a  13b  13c | Provide details of ethical approval (citing conduct of human research in accordance with the Declaration of Helsinki)  Summarize the research design (e.g., parallel trial/cross-over, randomized, counterbalanced, blinding, observational)  List the eligibility (inclusion/exclusion) criteria and sampling method  Characterize the study sample (e.g., demographics, anthropometry, lifestyle)  Report the setting/location and periods of recruitment and data collection  Justify the sample size (presenting the selected target effect size and error variances to replicate sample size estimates)  Detail all aspects of the groups/conditions (considering the need to verify the composition of ingested substances)  Define the pre-specified primary, secondary and/or mechanistic outcome variables  Rationalize the selection of test protocols, considering validity and reliability (e.g., coefficient of variation, familiarization)  Justify the smallest worthwhile effect or minimal clinically important difference  Detail the exact mechanisms of generating and concealing the random allocation sequence  Document whether participants and/or researchers were aware of allocation (e.g., exit questionnaire)  Describe within- and between-participant controls (e.g., replication/reporting of diet, physical activity, sleep, menstrual cycle)  Detail control of systematic influences of serial measurements (e.g., sequence effect in analysis model, wash-out interval)  Specify the contrast for primary inferences (i.e., relative to the appropriate control, not changes from baseline in each group/condition)  Clearly distinguish and fully justify any unplanned, interim or exploratory subgroup analyses  Describe any adjustments for violated statistical assumptions and for relevant covariates (e.g., baseline measures) | LN 98 – 100  LN 110 – 112  LN 102 – 108  LN 95 – 87  LN 101 – 102  LN 245 – 252  LN 126 – 145  LN 148 – 161  LN 185 – 188  LN 195 – 196  LN 204 – 205  LN 241 – 242  LN 264 – 268  LN148 – 151  LN 151 – 154  LN 120 – 122  LN 110 – 112  LN 275 – 276  LN 296 – 297  n/a  n/a |
| **Results**  *Participant flow*  *Outcomes* | 14a  14b  15a  15b  15c | Report the sample size at each phase from recruitment to analysis (with reasons for losses and exclusions)  Ensure data analysis matches research design, avoiding data pooling across groups/conditions (i.e., pseudoreplication  Report SI units and report measures of central tendency, variability, and effect size/precision (confidence intervals)  Report individual data/responses (e.g., draw figures showing the raw data in each group/condition) Document all relevant harms and unintended consequences observed | Figure 1  Figure 1  LN 275 – 312  Figures 4 and 5  n/a |
| **Discussion** | 16a  16b  16c | Present an objective and balanced interpretation of the observed data within the context of existing evidence  Consider the applicability and/or practical relevance of the research findings (e.g., external validity)  Acknowledge strengths and limitations of the research relevant to accurate interpretation (e.g., internal validity) | LN 315 – 434  n/a  LN 435 – 448 |
| **Other**  *Disclosures*  *Protocol* | 17  18 | State any relevant relationships (e.g., financial, technical, material support)  Identify any publicly registered or published protocol (explaining any deviations) | LN 459 – 460  n/a |

BETTS, J. A., GONZALEZ, J. T., BURKE, L. M., CLOSE, G. L., GARTHE, I., JAMES, L. J., JEUKENDRUP, A. E., MORTON, J. P., NIEMAN, D. C., PEELING, P., PHILLIPS, S. M., STELLINGWERFF, T., VAN LOON, L. J. C., WILLIAMS, C., WOOLF, K., MAUGHAN, R. & ATKINSON, G. 2020. PRESENT 2020: Text Expanding on the Checklist for Proper Reporting of Evidence in Sport and Exercise Nutrition Trials. *Int J Sport Nutr Exerc Metab,* 30**,** 2-13.
